# The role of contextualisation in enhancing non-communicable disease programmes and policy implementation to achieve health for all

**DOI:** 10.1186/s12961-020-00553-5

**Published:** 2020-04-17

**Authors:** Hueiming Liu, Mark D. Huffman, Kathy Trieu

**Affiliations:** 1 The George Institute for Global Health, University of New South Wales, Sydney, NSW Australia; 2 Department of Preventive Medicine and Center for Global Cardiovascular Health, Northwestern University’s Feinberg School of Medicine, Chicago, United States of America

**Keywords:** Non-communicable disease, policy analysis, context, health systems, fixed dose combination, sodium reduction

## Abstract

The September 2019 United Nations’ High-Level Meeting renewed political commitments to invest in universal health coverage by strengthening health systems, programmes and policies to achieve ‘health for all’. This Political Declaration is relevant to addressing the increasing global burden of non-communicable diseases, but how can evidence-based programmes and policies be meaningfully implemented and integrated into local contexts? In this Commentary, we describe how the process of contextualisation and associated tools, such as ecological frameworks, implementation research frameworks, health system indicators, effective system strengthening strategies and evidence mapping databases with priority-setting, can enhance the implementation and integration of non-communicable disease prevention and control policies and programmes. Examples across health platforms include (1) population approaches to reducing excess sodium intake, (2) fixed-dose combination therapy for cardiovascular disease prevention and control, and (3) health systems strengthening for improving the quality and safety of cardiovascular care. Contextualisation is needed to transfer evidence into locally relevant and impactful policies and programmes. The systematic and comprehensive use of contextualisation tools leverages key implementation research principles to achieve ‘health for all’.

## ‘Health for All’, non-communicable diseases and contextualisation

The United Nations Political Declaration on Universal Health Coverage on the 23^rd^ September 2019, garnered commitments from countries globally to strengthen the full spectrum of health services from prevention to treatment, rehabilitation and palliative care to achieve “*health for all*” [1]. This Political Declaration is directly relevant to implementing and integrating quality health service programmes and impactful policies to address non-communicable diseases (NCDs), which account for nearly two out of every three deaths globally, including the UN Sustainable Development Goal 3.4, “*to reduce the burden of premature mortality from NCDs by 1/3 by 2030*” [1]. However, in a 2020 report on global implementation of WHO’s NCD ‘best-buy’ policies demonstrated that, on average, just under half (49.3%) of these NCD-related policies had been implemented by 2017 in 151 countries [2]. Given these major gaps, how can evidence-based, NCD policies and programmes aligned with the Political Declaration be meaningfully integrated and implemented in local contexts?

The process of ‘contextualisation’ is necessary to allow for greater transferability of evidence across settings. We describe contextualisation as a way of incorporating local factors, embracing co-production with stakeholders using iterative research cycles, and building local research capacity. Contextualisation can be facilitated by the use of relevant tools, such as ecological frameworks, implementation research frameworks, health system indicators, effective system strengthening strategies, and evidence mapping databases with priority-setting. In this Commentary, we describe three examples of NCD prevention and control policies and programmes across health platforms to help achieve ‘health for all’, namely (1) population approaches to reducing excess dietary sodium intake, (2) fixed-dose combination therapy for cardiovascular disease prevention and control in primary healthcare, and (3) health systems strengthening for improving the quality and safety of NCD care at both outpatient and inpatient facilities.

## Population approaches to reducing excess dietary sodium intake

First, to address the estimated 3 million deaths attributable to excess dietary sodium intake worldwide, leading authoritative organisations recommend population-wide reductions in sodium consumption [3]. The WHO set a global sodium reduction target of 30% by 2025 to prevent and control NCDs through lowering blood pressure and recommended four sodium reduction strategies as “*best buys*” (described in the WHO technical package) [4]. While these approaches were developed based on effective interventions in a handful of countries, evidence of success in other countries has been more limited. A 2015 review identified 75 countries that had implemented national sodium reduction strategies; however, a Cochrane review conducted at the same time found that only five of ten countries had achieved decreased population sodium intake following the intervention [5]. Reasons for mixed results are likely due to differences in the contextual factors that affect implementation (e.g. funding, policy, organisation) and influence sodium intake, particularly differences in the sources of sodium in the diet.

This gap between research evidence and policy effectiveness was illustrated when the sodium reduction ‘best buys’ were implemented in Samoa (2013–2015), a vastly different context to the countries where the evidence of effective strategies originated (e.g. United Kingdom, Finland), and where null effects on population-level dietary sodium consumption were observed following programme implementation [6]. In Samoa, ‘best buy’ interventions, such as engagement with food manufacturers in voluntary product reformulation to contain less sodium, were not well accepted by government and the food industry. Another intervention, front-of-pack nutrition labelling, was not feasible to implement within the existing regulatory frameworks at the time [6]. Behaviour change communication and the establishment of supportive communities were more feasible. From the perspective of Story et al.’s ecological framework [7], which incorporates multi-level influences on what people consume, only individual and socioenvironmental factors were targeted in Samoa, and physical and macro-level environmental factors were insufficiently addressed. Furthermore, unlike diets in the United Kingdom or Finland, where most (>75%) dietary sodium intake came from processed or packaged foods, or diets in China, where 75% of dietary sodium intake came from salt added during cooking, diets in Samoa included both salt added by consumers and sodium added by manufacturers in processed foods as similar and major contributors [6]. This context meant that a multi-component strategy effectively targeting both consumers and food manufacturers was needed in Samoa. Therefore, two useful tools for co-developing or adapting effective sodium reduction strategies with stakeholders to suit the local context are (1) frameworks conceptualising the potential contextual factors that affect food and nutrient intake and implementation of nutrition interventions [7], and (2) collecting data on estimates of population-level dietary sources of sodium to design an effective, multi-level strategy, as developed by the WHO Regional Office for Europe [8].

## Fixed-dose combination therapy for cardiovascular disease prevention and control

The second example of contextualisation influencing implementation of NCD prevention and control comes from studies of fixed-dose combinations or polypill therapy. Polypills represent a strategy to implement cost-effective, multi-drug regimens for patients with or at high predicted risk for cardiovascular diseases [9]. In 2019, the PolyIran trial demonstrated that the combination of aspirin, atorvastatin, and either lisinopril or valsartan led to a 34% lower risk (95% CI 20–45%) for cardiovascular events compared with usual care over 5 years when implemented in a large, cluster-randomized trial across 236 villages (*n* = 6,838 participants) in Iran [10]. These results contrasted with the Kanyini-GAP polypill trial in Australia (*n* = 623 participants), where there were no significant differences in changes in risk factors nor clinical outcomes between groups over 18 months despite a 49% (95% CI 30–72%) higher relative rate of self-reported adherence to indicated therapy among individuals randomised to aspirin, simvastatin, lisinopril, and either atenolol or hydrochlorothiazide compared with usual care [11]. Differences in outcomes between these trials may be due to differences in the respective comparator groups, i.e. patients may have received different levels of usual care in Iran compared with Australia. In essence, the polypill model of care addressed different contextual problems in these trials – one that was related to access to essential medications in Iran compared with another to improve patient adherence and prescribing patterns in Australia. This contrast suggests that cross-sectional ‘snapshots’ of primary healthcare systems can provide researchers with useful information on how to tailor interventions and implementation strategies to meet local needs. For example, health system ‘vital sign’ profiling from the Primary Health Care Performance Initiative (https://improvingphc.org/vital-signs-profiles) serves as a useful contextualisation tool for primary care researchers and government policy-makers. This tool reports dashboard-style, country-level data on primary care financing, capacity, performance and equity that can influence polypill planning and uptake to reduce the burden of NCDs.

Despite established effectiveness across multiple trials, the use of polypills has not yet been widely adopted into practice and policy. Barriers in Australia were specific to pharmaceutical regulation, such as a lack of sufficient business case for the production of low-cost polypills and challenges to obtain marketing approval from peak regulatory bodies. In comparison, in a country like India, where polypills have been available in the market, their uptake has been limited by doctors’ perceptions that polypill combinations may be suboptimal, dosing options are too constrained or constraining, and polypill manufacturers may be less likely to adhere to Good Manufacturing Practices to produce high-quality combinations [12]. These differences illustrate the complex interplay between global and regional pharmaceutical companies, regulatory bodies, payers, national professional organisations and local health communities on how to incorporate polypills effectively for cardiovascular disease prevention and control.

Moving forward, systematic and comprehensive use of contextualisation tools is needed to address this complexity. For example, the Consolidated Framework for Implementation Research (cfirguide.org) helps to specify polypill combinations, actors engaged in polypill implementation (e.g. patients, physicians), modifiable and non-modifiable inner and outer contexts (e.g. regulatory pathways for commercialisation, bulk pre-purchasing by government payers), and the process of polypill implementation and normalisation into clinical practice (e.g. incorporating into clinical practice guidelines). Second, the WHO technical package for cardiovascular disease management in primary care includes an ‘Access to essential medicines and technology’ module that can be used to map stakeholders at the national, subnational and primary care levels and to identify suitable strategies, including the potential role of the polypill to improve access to medicines [13].

## Health systems strengthening for improving the quality and safety of NCD care

Third, to improve quality and safety of NCD care, including at both outpatient and inpatient settings, health system strengthening must be central to research, practice and policy. Low- and middle-income health systems are often insufficiently resourced to provide optimal care for cardiovascular conditions. The 2018 Health Care Provider Performance Review, whose objective was to synthesise evidence about the most effective ways to improve healthcare provider performance, included 670 reports from 337 studies of 118 strategies, including 13 strategy groups, across a range of interventions, training types and countries [14]. The review identified the two most effective strategy categories among the 13 groups, namely (1) group problem solving, which led to a 14% larger marginal effect of improving provider performance compared with other interventions, and (2) training, which led to a 6% larger marginal effect. The authors also identified contextual factors that were associated with larger effect sizes. First, for every 1% higher baseline care, there was a 0.2% smaller effect of quality improvement interventions, suggesting that healthcare providers and facilities with lower performing metrics are better targets for intervention. Another contextual factor identified was that studies exclusively performed in public health facilities had a 7% larger effect. Therefore, researchers, practitioners and policy-makers should focus on the most challenging problems with the widest performance gaps in public settings to improve quality, safety and equity.

Data from the Health Care Provider Performance Review are available (www.hcpperformancereview.org), which represents as a useful contextualisation tool to examine disaggregated results by country, intervention and outcomes of interest for future programme planning. Similarly, Palagyi et al. [15] mapped evidence of primary care interventions in the Asia Pacific region, which included a summary of both intervention effectiveness and implementation by country setting. This evidence map was based on expert advice, policy analysis, synthesis of academic literature and Delphi surveys with key informants.

These databases help policy-makers prioritise interventions that would be more likely to work in their local context.

## Conclusion

Contextualisation is a vital pathway to translate robust evidence into local programmes and polices needed to achieve universal health coverage goals, which UN Member States will begin to benchmark in 2020 toward their 2030 targets. Tools for contextualisation can facilitate systematic and comprehensive approaches for local stakeholders to identify key needs for programmes and policies to be effective, implemented and integrated across health platforms to help achieve ‘health for all’.

## Data Availability

Not applicable.
